# Thoracoabdominal Normothermic Regional Perfusion Attenuates the Protective Effect of Hypothermic Machine Perfusion on Delayed Graft Function in DCD Kidney Transplantation

**DOI:** 10.1155/joot/8082011

**Published:** 2026-10-02

**Authors:** Ayham Asassfeh, Jiro Kimura, Enida Luma, Matthew Harris, Emre Arpali, Ty B. Dunn, Matthew Cooper, Badi Rawashdeh

**Affiliations:** ^1^ Division of Transplant Surgery Medical College of Wisconsin Milwaukee Wisconsin USA mcw.edu; ^2^ Medical College of Wisconsin Milwaukee Wisconsin USA mcw.edu

## Abstract

**Background:**

Thoracoabdominal normothermic regional perfusion (TA‐NRP) and hypothermic machine perfusion (HMP) reduce ischemia–reperfusion injury after donation after circulatory death (DCD) kidney transplantation, but their combined effects remain unclear.

**Methods:**

Adult DCD kidney‐only recipients undergoing pretransplant dialysis in the OPTN registry (2020–2025) were stratified by TA‐NRP and HMP. The primary outcome was delayed graft function (DGF). Multivariable logistic regression included a TA‐NRP × HMP interaction and adjusted for recipient and donor factors. Death‐censored graft survival (DCGS) was evaluated by Kaplan–Meier analysis.

**Results:**

Among 3502 recipients, DGF differed by preservation strategy. In the non‐TA‐NRP cohort, HMP significantly reduced DGF compared with static cold storage (SCS) (44.2% vs 53.8%; *p* = 0.001) and remained independently protective (adjusted OR 0.56; 95% CI 0.44–0.71; *p* < 0.001). In the TA‐NRP cohort, DGF rates were similar between HMP and SCS (26.7% vs 27.0%; *p* = 0.98), with no independent association after adjustment (adjusted OR 0.82; 95% CI 0.62–1.08; *p* = 0.16). A significant TA‐NRP × HMP interaction was observed (*p* = 0.039). In the absence of HMP, TA‐NRP was strongly protective against DGF (adjusted OR 0.30; 95% CI 0.22–0.42; *p* < 0.001). At 3 years, DCGS was 93.9% (95% CI 91.5–96.4) for TA‐NRP‐HMP, 94.3% (90.9–97.9) for TA‐NRP‐SCS, 93.5% (91.5–95.6) for non‐TA‐NRP‐HMP, and 92.7% (89.1–96.6) for non‐TA‐NRP‐SCS, with no difference between groups (log‐rank *p* = 0.73).

**Conclusion:**

TA‐NRP was associated with lower DGF than conventional DCD procurement. Within the TA‐NRP cohort, HMP was not associated with additional reductions in DGF. These findings should be interpreted in light of the surrogate definition used to identify TA‐NRP in the OPTN registry and warrant prospective validation.

## 1. Introduction

Donation after circulatory death (DCD) kidney transplantation has become a critical strategy to address the persistent organ shortage [1, 2]. However, DCD grafts remain disproportionately affected by delayed graft function (DGF), a complication that decreases graft utilization and increases resource utilization, prolongs hospitalization, and adversely impacts long‐term graft survival [3–5]. Warm ischemic injury during circulatory arrest is the primary driver of DGF in DCD transplantation, prompting widespread adoption of preservation strategies aimed at mitigating ischemia–reperfusion injury [6].

Among these approaches, thoracoabdominal normothermic regional perfusion (TA‐NRP) and hypothermic machine perfusion (HMP) have gained increasing attention. TA‐NRP restores in situ oxygenated circulation after death declaration, allowing metabolic resuscitation, adenosine triphosphate (ATP) replenishment, and endothelial stabilization prior to procurement [7, 8]. In contrast, HMP is applied after organ recovery and primarily targets ischemic injury during cold preservation through continuous perfusion and improved microvascular preservation [9, 10].

Although both strategies have independently been associated with lower rates of DGF, their interaction has not been systematically evaluated. Mechanistically, if TA‐NRP reverses a substantial component of warm ischemic injury before cold storage, the incremental benefit of HMP may be attenuated. Conversely, in conventional DCD recovery without TA‐NRP, HMP may play a critical compensatory role. Clarifying whether these strategies are synergistic, redundant, or context‐dependent has direct implications for preservation protocols, allocation logistics, and cost‐effectiveness in modern DCD transplantation.

In this contemporary national cohort study, we examined the association between HMP and DGF in DCD kidney transplantation stratified by TA‐NRP utilization. We hypothesized that the protective effect of HMP would be significantly attenuated in kidneys recovered with TA‐NRP compared with conventional DCD procurement.

## 2. Methods

### 2.1. Data and Study Design

We performed a retrospective cohort study using the Organ Procurement and Transplantation Network registry to identify adult (≥ 18 years) dialysis‐dependent recipients of deceased‐donor kidney‐only transplants between January 1, 2020, and December 31, 2025. Exclusions included pre‐emptive (nondialysis‐dependent) recipients, living‐donor recipients, prior kidney transplant recipients, recipients with missing DGF data, and recipients of DCD kidneys from donors whose heart was neither transplanted nor recovered for transplantation.

Because direct TA‐NRP capture in OPTN was not available, TA‐NRP utilization was identified using a previously described surrogate based on donor heart disposition and agonal‐to‐clamp time, reflecting TA‐NRP [11–15]. Within the restricted DCD cohort, agonal‐to‐clamp time was used to distinguish procurement strategy. Durations ≤ 30 min were classified as non‐TA‐NRP DCD because this interval would generally be insufficient to accommodate both establishment of NRP and the subsequent period of regional perfusion before aortic cross‐clamping. Durations ≥ 40 min were classified as TA‐NRP DCD because they are more consistent with the expected temporal profile of TA‐NRP. The intermediate interval (31–39 min) was excluded to minimize exposure misclassification. The distribution and counts of agonal‐to‐clamp times are presented in a supporting figure. Recipients were then stratified according to donor TA‐NRP status and use of HMP (Figure 1). In accordance with institutional policy, this study was determined to be exempt from Institutional Review Board review because it involved analysis of de‐identified registry data. Because the study used existing de‐identified data and involved no direct participant contact, informed consent was waived.

**FIGURE 1:**
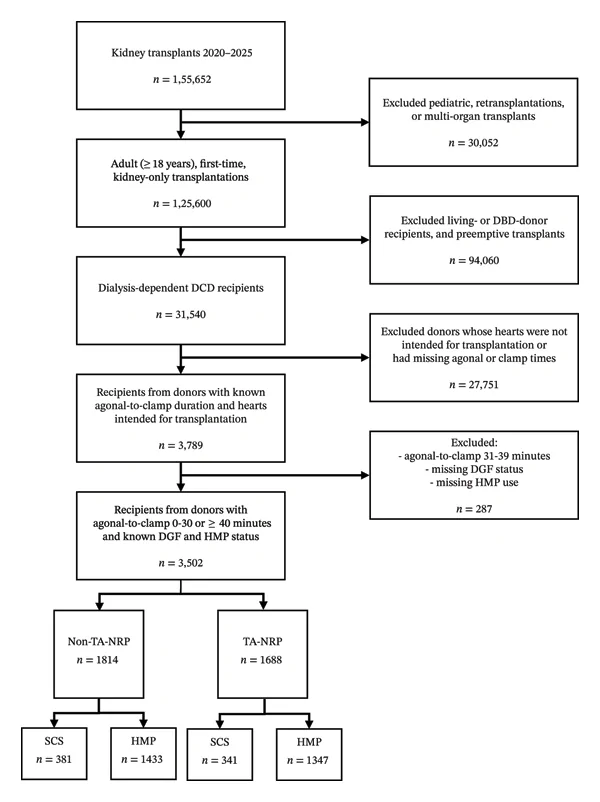
Flow diagram. Legend: DCD, donation after circulatory death; TA‐NRP, normothermic regional perfusion; HMP, hypothermic machine perfusion; SCS, static cold storage; DGF, delayed graft function; OPTN, organ procurement and transplantation network.

### 2.2. Outcomes

The primary outcome was DGF, defined as dialysis within the first 7 days after transplantation. Unadjusted DGF incidence was reported across the four preservation groups. The secondary outcome was death‐censored graft survival (DCGS), defined as time from transplantation to graft failure or re‐transplantation, with death and loss to follow‐up treated as censoring events. DCGS was evaluated at 1 and 3 years after transplantation.

### 2.3. Statistical Analysis

Baseline characteristics were compared across preservation strategies. Continuous variables were summarized as medians with interquartile ranges and compared using the Kruskal–Wallis test. Categorical variables were summarized as counts with percentages and compared using Pearson’s chi‐square test.

Adjusted odds ratios (ORs) for DGF were estimated using multivariable logistic regression in the full cohort, including TA‐NRP, HMP, and a TA‐NRP × HMP interaction term, adjusting for recipient age (per 5 years increase), recipient sex, recipient diabetes, donor KDPI (per 10% increase), and cold ischemia time (per 6 h increase). DCGS was evaluated using Kaplan–Meier methods and compared using the log‐rank test. All statistical analyses were performed using R (Version 4.5.1).

## 3. Results

### 3.1. Study Population

A total of 3502 adult DCD kidney transplant recipients met inclusion criteria and were stratified by donor TA‐NRP status and preservation method (HMP vs SCS) into four groups: non‐TA‐NRP with HMP (*n* = 1433), non‐TA‐NRP with SCS (*n* = 381), TA‐NRP with HMP (*n* = 1347), and TA‐NRP with SCS (*n* = 341).

### 3.2. Baseline Characteristics

Baseline characteristics for the TA‐NRP and non‐TA‐NRP cohorts are shown in Table 1.

**TABLE 1:** Baseline characteristics of recipients of TA‐NRP donor kidneys stratified by pump use.

Group	TA‐NRP			Non‐TA‐NRP		
Characteristic	HMP 1347 (79.8%)	SCS 341 (20.2%)	*p*	HMP 1433 (79%)	SCS 381 (21%)	*p*
*Recipient characteristics*						
Age, years	50.0 (40.0–61.0)	46.0 (36.0–57.0)	< 0.001	52.0 (40.0–62.0)	47.0 (38.0–57.0)	< 0.001
Sex			0.12			0.20
Female	517 (38%)	147 (43%)		567 (40%)	166 (44%)	
Male	830 (62%)	194 (57%)		866 (60%)	215 (56%)	
Race/ethnicity			0.20			0.70
White	385 (29%)	82 (24%)		387 (27%)	98 (26%)	
African American	521 (39%)	142 (42%)		607 (42%)	164 (43%)	
Asian	77 (5.8%)	15 (4.4%)		84 (5.9%)	23 (6.0%)	
Hispanic/Latino	311 (23%)	94 (28%)		308 (22%)	89 (23%)	
Other	44 (3.3%)	8 (2.3%)		44 (3.1%)	7 (1.8%)	
Diabetic	479 (36%)	88 (26%)	< 0.001	491 (34%)	120 (31%)	0.30
Recipient ABO			0.026			0.11
O	741 (55%)	200 (59%)		826 (58%)	195 (51%)	
A	436 (32%)	96 (28%)		393 (27%)	124 (33%)	
B	134 (9.9%)	43 (13%)		169 (12%)	52 (14%)	
AB	36 (2.7%)	2 (0.6%)		45 (3.1%)	10 (2.6%)	
BMI	28.7 (24.6–33.1)	27.9 (23.8–32.8)	0.091	28.8 (25.1–33.3)	27.8 (23.7–32.4)	0.007

*Donor characteristics*						
Donor sex			0.15			0.13
Female	283 (21%)	59 (17%)		289 (20%)	63 (17%)	
Male	1064 (79%)	282 (83%)		1144 (80%)	318 (83%)	
Donor race/ethnicity			0.006			0.20
White	1038 (77%)	239 (70%)		1075 (75%)	281 (74%)	
Black	104 (7.7%)	27 (7.9%)		144 (10%)	44 (12%)	
Hispanic/Latino	153 (11%)	64 (19%)		167 (12%)	42 (11%)	
Asian	20 (1.5%)	6 (1.8%)		19 (1.3%)	10 (2.6%)	
Other	32 (2.4%)	5 (1.5%)		28 (2.0%)	4 (1.0%)	
KDPI	29.0 (19.0–40.0)	23.0 (16.0–34.0)	< 0.001	27.0 (18.0–40.0)	22.0 (15.0–31.0)	< 0.001
Donor diabetes			0.60			0.008
No	1297 (96%)	330 (97%)		1372 (96%)	370 (97%)	
Yes	46 (3.4%)	9 (2.6%)		58 (4.0%)	7 (1.8%)	
Unknown	4 (0.3%)	2 (0.6%)		3 (0.2%)	4 (1.0%)	
Donor ABO			0.20			0.20
O	753 (56%)	204 (60%)		850 (59%)	202 (53%)	
A	476 (35%)	102 (30%)		437 (30%)	133 (35%)	
B	107 (7.9%)	33 (9.7%)		132 (9.2%)	42 (11%)	
AB	11 (0.8%)	2 (0.6%)		14 (1.0%)	4 (1.0%)	
Donor BMI	26.9 (23.6–30.7)	27.0 (23.3–30.7)	0.80	27.4 (24.0–32.4)	26.2 (22.9–30.1)	< 0.001

*Transplant characteristics*						
Cold ischemia time, hours	19.8 (16.0–23.5)	17.2 (13.7–21.0)	< 0.001	19.9 (16.6–23.2)	17.5 (12.5–21.5)	< 0.001
Distance, miles	99.0 (15.0–199.0)	135.0 (35.0–211.0)	0.011	104.0 (22.0–194.0)	139.0 (48.0–221.0)	< 0.001
Waitlist time, months	10.1 (2.1–32.8)	13.6 (2.6–40.5)	0.015	12.1 (2.3–38.7)	11.3 (2.7–42.0)	0.70

*Note:* Values are presented as *n* (%) or median [interquartile range], Table 1 includes baseline characteristics stratified separately within the TA‐NRP and non‐TA‐NRP cohorts by preservation method (HMP vs SCS), *p* values were calculated for comparisons between HMP and SCS within each cohort using Pearson’s chi‐square test for categorical variables and Kruskal–Wallis test for continuous variables, ABO, blood group.

Abbreviations: BMI, body mass index; HMP, hypothermic machine perfusion; KDPI, kidney donor profile index; SCS, static cold storage.

In the TA‐NRP cohort, HMP was used in 1347 (79.8%) transplants and SCS in 341 (20.2%). Compared with SCS, HMP recipients were older (median 50.0 vs. 46.0 years, *p* < 0.001) and more frequently diabetic (36% vs. 26%, *p* < 0.001). HMP kidneys had higher KDPI (29.0 vs. 23.0, *p* < 0.001) and longer cold ischemia time (19.8 vs. 17.2 h, *p* < 0.001), while transport distance and waitlist time were shorter (both *p*  <  0.05).

In the non‐TA‐NRP cohort, HMP was used in 1433 (79%) transplants and SCS in 381 (21%). HMP recipients were older (median 52.0 vs. 47.0 years, *p* < 0.001). HMP kidneys had higher KDPI (27.0 vs. 22.0, *p* < 0.001), higher donor body mass index (BMI) (27.4 vs. 26.2 kg/m^2^, *p* < 0.001), and longer cold ischemia time (19.9 vs. 17.5 h, *p* < 0.001). Donor diabetes was more frequent, and transport distance was shorter in the HMP group (both *p* ≤ 0.01).

### 3.3. DGF

DGF incidence differed by preservation strategy (Figure 2). Among non‐TA‐NRP kidneys, HMP was associated with a significantly lower incidence of DGF compared with SCS (44.2% vs 53.8%; *p* = 0.0011). In contrast, within the TA‐NRP cohort, DGF rates were similar between HMP and SCS (26.7% vs 27.0%; *p* = 0.98).

**FIGURE 2:**
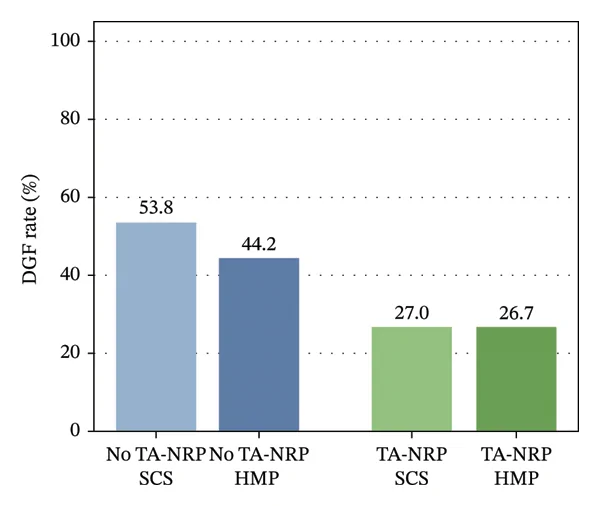
Unadjusted DGF rates by TA‐NRP use and preservation strategy. Legend: TA‐NRP, normothermic regional perfusion; HMP, hypothermic machine perfusion; SCS, static cold storage; DGF, delayed graft function.

In the adjusted analysis (Figure 3, Table 2), a significant interaction was observed between TA‐NRP and HMP (interaction OR 1.46; 95% CI 1.02–2.09; *p* = 0.039). Among non‐TA‐NRP kidneys, HMP was independently associated with lower odds of DGF (adjusted OR 0.56; 95% CI 0.44–0.71; *p*  <  0.001), whereas in the TA‐NRP cohort, HMP was not significantly associated with DGF (adjusted OR 0.82; 95% CI 0.62–1.08; *p* = 0.16). In the absence of HMP, TA‐NRP was strongly protective against DGF (adjusted OR 0.30; 95% CI 0.22–0.42; *p*  <  0.001).

**FIGURE 3:**
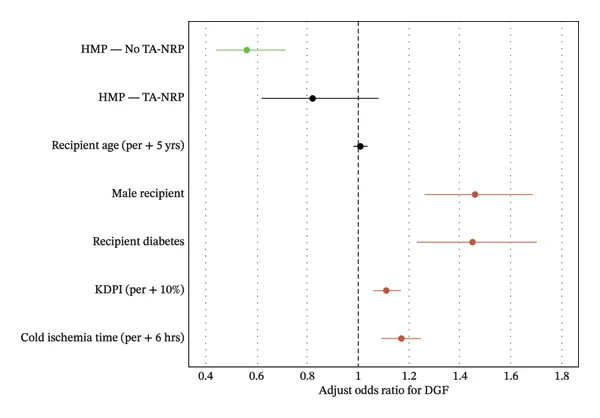
Multivariable adjusted odds ratios for delayed graft function. Legend: HMP, hypothermic machine perfusion; TA‐NRP, normothermic regional perfusion; DGF, delayed graft function; KDPI, kidney donor profile index; OR, odds ratio.

**TABLE 2:** Adjusted multivariable analysis for DGF by TA‐NRP and preservation strategy.

Variable	OR (95% CI)	*p* value
TA‐NRP × HMP interaction	1.46 (1.02–2.09)	**0.039**
HMP vs SCS in non‐TA‐NRP kidneys	0.56 (0.44–0.71)	**<** **0.001**
HMP vs SCS in TA‐NRP kidneys	0.82 (0.62–1.08)	0.16
NRP vs non‐TA‐NRP without HMP	0.30 (0.22–0.42)	**<** **0.001**
Recipient age (per 5 years increase)	1.01 (0.98–1.04)	0.50
Male recipient	1.46 (1.26–1.69)	**<** **0.001**
Recipient diabetes	1.45 (1.23–1.70)	**<** **0.001**
KDPI (per 10% increase)	1.11 (1.06–1.17)	**<** **0.001**
Cold ischemia time (per 6 h increase)	1.17 (1.09–1.25)	**<** **0.001**

*Note:* Odds ratios were estimated using multivariable logistic regression for delayed graft function (DGF), including a TA‐NRP × HMP interaction term and adjustment for recipient age, sex, diabetes, kidney donor profile index (KDPI), and cold ischemia time, TA‐NRP, thoracoabdominal normothermic regional perfusion. Bold values are statistically significant with p values equal to or less than 0.05.

Abbreviations: CI, confidence interval; DGF, delayed graft function; HMP, hypothermic machine perfusion; KDPI, kidney donor profile index; OR, odds ratio; SCS, static cold storage.

Male recipient sex (OR 1.46; 95% CI 1.26–1.69; *p*  <  0.001) and recipient diabetes (OR 1.45; 95% CI 1.23–1.70; *p*  < 0.001) were associated with higher odds of DGF. Cold ischemia time was associated with increased odds of DGF (OR 1.17 per 6 h increase; 95% CI 1.09–1.25; *p*  <  0.001), as was KDPI (OR 1.11 per 10% increase; 95% CI 1.06–1.17; *p*  <  0.001). Recipient age was not associated with DGF (OR 1.01 per 5 year increase; 95% CI 0.98–1.04; *p* = 0.50).

### 3.4. DCGS

At 1 year, DCGS was 98.3% (95% CI 97.5–99.1) for TA‐NRP‐HMP, 97.8% (96.2–99.4) for TA‐NRP‐SCS, 96.9% (95.9–97.9) for non‐TA‐NRP‐HMP, and 97.6% (96.0–99.3) for non‐TA‐NRP‐SCS (Figure 2). At 3 years, DCGS was 93.9% (91.5–96.4), 94.3% (90.9–97.9), 93.5% (91.5–95.6), and 92.7% (89.1–96.6), respectively, with no significant difference between groups (log‐rank *p* = 0.73). (Figure 4).

**FIGURE 4:**
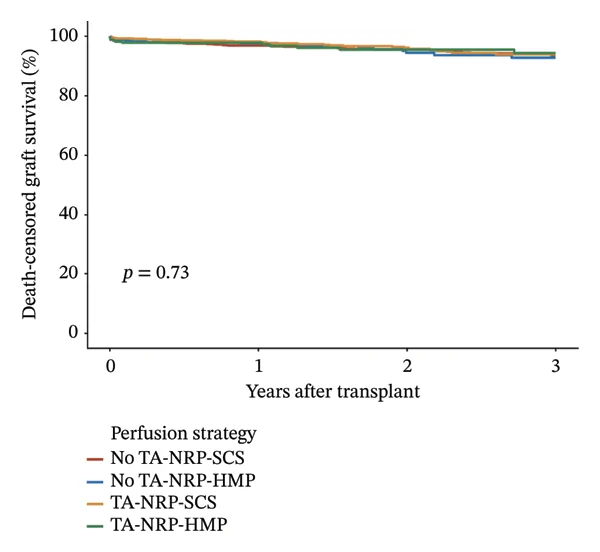
Three‐year death‐censored graft survival by TA‐NRP status and preservation method. Legend: TA‐NRP, normothermic regional perfusion; HMP, hypothermic machine perfusion; SCS, static cold storage.

## 4. Discussion

In this contemporary national cohort of DCD kidney transplant recipients, the effect of HMP on DGF was strongly modified by procurement strategy. In conventional DCD recovery without TA‐NRP, HMP was independently associated with a substantial reduction in DGF, whereas in kidneys recovered with TA‐NRP this association was attenuated and no longer statistically significant. These findings suggest that the benefit of HMP is not uniform but depends on the degree to which warm ischemic injury has already been mitigated at the time of procurement.

DGF in DCD transplantation is primarily driven by warm ischemia sustained during circulatory arrest, resulting in ATP depletion, endothelial dysfunction, microvascular congestion, and heightened ischemia–reperfusion injury [14, 16]. TA‐NRP restores in situ oxygenated blood flow prior to procurement, facilitating metabolic resuscitation and endothelial stabilization [17]. By partially reversing warm ischemic injury before cold preservation, TA‐NRP may reduce the biological substrate upon which HMP exerts its protective effects [18]. An alternative explanation is that a subset of DCD kidneys may sustain severe ischemic injury before procurement, resulting in irreversible acute tubular injury despite the use of advanced preservation strategies. In this context, both TA‐NRP and HMP may reach the limits of their biological capacity to improve early graft function, resulting in similar DGF rates among kidneys recovered with TA‐NRP regardless of subsequent machine perfusion. Future studies incorporating procurement biopsy findings, perfusion characteristics, and biomarkers of organ injury may help identify kidneys most likely to benefit from additional preservation strategies.

This mechanistic framework is consistent with our findings: when warm injury remains uncorrected, HMP provides substantial protection, whereas after TA‐NRP its incremental benefit appears limited. The marked separation in DGF rates between TA‐NRP and non‐TA‐NRP groups also supports the face validity of the surrogate definition, as the observed patterns align with expected biological differences in warm ischemic injury.

The protective association of HMP in the non‐TA‐NRP cohort persisted despite preferential use in grafts with higher KDPI and longer cold ischemia times, supporting a true compensatory role in higher‐risk organs and strengthening the robustness of the observed effect. In contrast, in the TA‐NRP cohort, unadjusted DGF rates were nearly identical between pumped and nonpumped kidneys, and adjusted analyses showed no significant independent association, further supporting attenuation rather than confounding as the explanation for the interaction observed.

Despite these differences in early graft function, DCGS remained uniformly high across all preservation strategies, exceeding 96% at 1 year and 92% at 3 years. This suggests that early functional advantages did not translate into measurable mid‐term graft survival differences within the available follow‐up, likely reflecting contemporary improvements in management, immunosuppression, and the overall favorable outcomes of modern DCD transplantation [19, 20].

Recipient and donor characteristics continued to contribute independently to DGF risk in expected directions, including recipient age, male sex, diabetes, KDPI, and cold ischemia time, underscoring the multifactorial nature of early graft dysfunction even in the setting of evolving procurement and preservation techniques.

These findings may have clinical and operational implications. HMP is resource intensive, logistically complex, and associated with additional cost. If its incremental benefit is limited when TA‐NRP is used, the routine application of both technologies to the same graft may warrant further evaluation. A more selective approach in which HMP is prioritized for conventional DCD recovery could potentially improve cost efficiency while maintaining favorable outcomes. As TA‐NRP adoption continues to expand, tailoring preservation approaches to procurement context rather than applying them universally may represent a more efficient use of resources. Formal cost‐effectiveness analyses are needed to further evaluate these strategies.

Several limitations merit consideration. As a registry‐based analysis, residual confounding cannot be excluded, particularly with respect to center‐level practice patterns and selection in pump utilization. Because OPTN does not directly capture TA‐NRP use, we identified TA‐NRP using a previously published surrogate based on donor heart disposition and agonal‐to‐clamp time. Although this approach has been validated in prior national registry analyses, some misclassification is unavoidable, particularly because agonal‐to‐clamp time may overlap between conventional DCD procurement and TA‐NRP in selected cases. Consequently, our findings should be interpreted as associations rather than definitive comparisons of procurement techniques. Detailed perfusion parameters, duration of TA‐NRP, and biochemical measures of organ quality were not captured. In addition, longer‐term graft outcomes beyond 3 years were not evaluated and may reveal differential effects not apparent in the mid‐term. Nevertheless, the large contemporary sample size and stratified national analysis provide robust real‐world insight into the interaction between procurement and preservation strategies.

In conclusion, in this contemporary national cohort of DCD kidney transplant recipients, TA‐NRP was associated with substantially lower rates of DGF compared with conventional DCD procurement. Within the TA‐NRP cohort, HMP was not associated with a further reduction in DGF, whereas HMP remained independently associated with lower DGF in conventional DCD procurement. These findings suggest that the clinical benefit of HMP may vary according to the procurement strategy and support further prospective studies to define the optimal preservation approach and evaluate its clinical and cost‐effectiveness in DCD kidney transplantation.

## Funding

This project received no funding.

## Disclosure

The data reported here have been supplied by the United Network for Organ Sharing (UNOS) as the contractor for the Organ Procurement and Transplantation Network (OPTN). The interpretation and reporting of these data are the responsibility of the authors and do not necessarily reflect the views or policies of the OPTN, UNOS, or the U.S. Government.

## Conflicts of Interest

The authors declare no conflicts of interest.

## Supporting Information

Additional supporting information can be found online in the Supporting Information section.

## Supporting information


**Supporting Information** Supporting Figure 1. Distribution and counts of agonal‐to‐clamp times among the study cohort.

## Data Availability

The data that support the findings of this study are available from UNOS/OPTN. Restrictions apply to the availability of these data, which were used under license for this study. Data are available from the authors with the permission of UNOS/OPTN.
